# Health care utilization following the adoption of U.S. paid sick leave mandates: a cohort study using health insurance claims data

**DOI:** 10.1016/j.lana.2025.101174

**Published:** 2025-07-07

**Authors:** Kevin Callison, Michael F. Pesko, Serena Phillips, Julie Ann Sosa

**Affiliations:** a Department of Health Policy and Management, Celia Scott Weatherhead School of Public Health, Murphy Institute of Political Economy, Tulane University, 1440 Canal St., Suite 1900, New Orleans, LA, 70112, United States; b Department of Economics, College of Arts and Science, University of Missouri, 615 Locust St., Columbia, MO, 65211, United States; c Department of Surgery, School of Medicine, University of California San Francisco (UCSF), 513 Parnassus Ave., MSB, #320C, San Francisco, CA, 94143, United States

**Keywords:** Paid sick leave, Health care utilization, Health policy

## Abstract

**Background:**

The U.S. is one of the only developed countries in the world without a federal requirement that employers provide paid sick leave (PSL) to workers. We evaluated the association between state and local PSL mandates and health care utilization among U.S. workers.

**Methods:**

We conducted a cohort analysis using administrative health insurance claims for 2.3 million private sector workers aged 40 to 64 from 2011 through 2019. Difference-in-differences models compared health care utilization before and after PSL mandate enactment between workers in areas with and without mandated PSL coverage. Outcomes included visits to primary care physicians (PCP), specialists, preventive care, diagnostic services, emergency department, urgent care visits, and hospitalizations.

**Findings:**

PSL mandates were associated with an increased probability of a past year PCP visit (4.79pp; 95% CI, 1.39–8.19), specialist visit (2.71pp, 95% CI, 0.98–4.44), preventive care visit (2.75pp; 95% CI, −0.36 to 5.86), and outpatient diagnostic visits (2.20pp, 95% CI, 1.21–3.19). PSL mandates were also associated with increases in the average annual number of specialist, preventive care, outpatient diagnostic, and urgent care visits. Estimates were generally larger for those working in industries that have historically maintained low rates of PSL coverage in the U.S.

**Interpretation:**

PSL mandates were associated with greater use of PCP, specialist, diagnostic, and preventive care services. These findings highlight the role for policies that enhance workplace flexibility, including PSL, to improve access to health care services.

**Funding:**

The research was supported by grant R01CA237888 from the 10.13039/100000054National Cancer Institute.

**Research in context**

**Evidence before this study**

Previous studies have examined the association between paid sick leave (PSL) coverage and health care utilization, finding that PSL is associated with greater likelihood of accessing care, particularly among workers with otherwise limited access to paid leave. However, much of this literature relies on cross-sectional comparisons between workers with and without PSL coverage, making it difficult to rule out confounding due to unobserved differences between these groups. Studies that leverage stae and local PSL mandates in the U.S. as natural experiments have provided stronger causal evidence but often focus on single states or narrow sets of outcomes, limiting generalizability.

**Added value of this study**

This study uses a large-scale, multi-state sample of privately insured workers aged 40–64 years and applies a difference-in-differences framework to estimate the association between PSL mandates and healthcare utilization in the U.S. By addressing selection bias through quasi-experimental methods, we provide the most robust evidence to date on the impact of PSL coverage on healthcare utilization. Our findings indicate that PSL mandates were associated with modest increases in the likelihood of accessing primary and specialist care, preventive services, and outpatient diagnostic services, with larger effects observed among workers in U.S. industries with historically low PSL coverage.

**Implications of all the available evidence**

Our findings provide robust evidence that PSL mandates increase healthcare utilization, especially among workers in industries with lower pre-mandate PSL coverage. This evidence suggests that PSL may help reduce barriers to healthcare and encourage care-seeking among workers who might otherwise forego medical visits. These findings highlight the importance of PSL in promoting access to health services and improving health outcomes for workers, particularly in contexts where healthcare utilization has been historically constrained. Given the widespread adoption of paid sick leave mandates worldwide, this study offers valuable insights for policymakers interested in understanding the broader health impacts of such policies.

## Introduction

Work commitments, time constraints, and financial hardship represent barriers to health care access and contribute to the underutilization of health care services.^1^^,^^2^ Paid sick leave (PSL) can address these barriers by increasing workplace flexibility and alleviating concerns about lost wages when individuals miss work due to illness or to seek care.

The Families First Coronavirus Response Act of 2020 provided up to two weeks of emergency PSL for COVID-19-related illness for private sector workers at firms with fewer than 500 employees.^3^ However, this benefit expired at the end of 2020, and the U.S. remains one of the only developed countries in the world with no federal requirement that employers provide PSL to their workers.^4^ Since 2007, 14 states, Washington D.C., and several municipalities in the U.S. have mandated employer-sponsored PSL, expanding coverage to more than 20 million workers.^5^^,^^6^ Still, one quarter of private sector workers in the U.S. and nearly half of all workers in the bottom quartile of the earnings distribution lacked PSL coverage in 2022.^7^^,^^8^

Studies generally report positive associations between PSL and health care utilization, including physician visits,^9^^,^^10^ preventive care,^9^^,^^11^, ^12^, ^13^, ^14^, ^15^, ^16^, ^17^ and outpatient services,^18^^,^^19^ with some evidence indicating PSL is associated with fewer emergency department (ED) visits.^10^^,^^20^^,^^21^ However, with few exceptions, these studies either rely on adjusted comparisons between those with and without PSL coverage, raising concerns over potential bias from unobserved confounders, or on single-state settings, limiting generalizability. Results from studies using more rigorous research designs have been less definitive, with some finding positive associations between PSL and clinical encounters^14^^,^^21^, ^22^, ^23^ and others finding mixed^10^ or no statistically significant associations.^24^

This study represents the first large-scale analysis of the association between PSL coverage and health care utilization, including physician and specialist visits, visits for preventive care and outpatient diagnostic services, urgent care and ED use, and hospital stays, for private sector U.S. workers leveraging the plausibly exogenous variation in coverage from the adoption of state and local PSL mandates.

## Methods

### Data and sample

We used data from the Merative™ MarketScan® Research Databases to track health care utilization and insurance enrolment for working adults with employer-sponsored coverage from 2011 through 2019. Data were collected from large, private sector employers' and private health insurance plans’ administrative claims for employer-sponsored plans. This study was deemed to be exempt from review by the institutional review board at Tulane University.

We restricted our sample to primary policy holders (not a spouse or dependent) between the ages of 40 and 64 years who were continuously enrolled in the same health plan for a full calendar year. The age range was informed by three considerations: first, middle-age and older workers use more health care services than younger workers (workers ages 40 and above during our sample period were 13% more likely to have at least one clinician visit in the past year and 24% more likely to have more than one visit compared to younger workers), and thus may be more responsive to changes in PSL coverage; second, many routine preventive care services are recommended for those ages 40 and older; and third, the majority of individuals in this age range are active in the labour force and could benefit directly from expanded PSL coverage. The continuous enrolment requirement ensured that we avoided instances where individuals sought care during periods when they were not covered by an insurance plan in our data, preventing us from observing their health care utilization. These restrictions resulted in a sample of approximately 2.3 million private-sector workers per year, with each worker observed for an average of 5.2 years.

We aggregated the data to the metropolitan statistical area (MSA) level, the geographic unit available in the MarketScan® data, calculated MSA-year average age and share female, and added data on race, ethnicity, and educational attainment for working adults ages 40 to 64 with employer-sponsored insurance coverage from the American Community Survey.^25^ To this, we merged data from the University of Kentucky Center for Poverty Research that included state-level unemployment and uninsured rates to address potential clinical capacity constraints associated with Affordable Care Act Medicaid expansions.^26^ For MSAs that spanned multiple states, we created MSA-state pairs to ensure that each portion of an MSA was assigned to the correct policy environment based on its state. This approach prevents misclassification of treatment status for MSAs where only part of the area was subject to a PSL mandate. For simplicity, we refer to these as MSAs rather than MSA-state pairs throughout the paper. Our final sample included 247 MSAs, 62 exposed to a PSL mandate, observed over nine years for a total of 2223 observations. We followed the STROBE reporting guidelines for cohort studies (see Appendix Table A16).

### Paid sick leave coverage

We constructed a binary measure of PSL mandate exposure equal to 1 in the year that workers in an MSA were exposed to either a state, county, or city mandate and equal to 0 otherwise. We excluded MSAs exposed to a PSL mandate before 2015 to establish a baseline for comparing outcome trends prior to mandate enactment. See Appendix Table A1 for details on the mandates included in our analysis.

### Outcomes

We examined several categories of health care utilization classified by procedure and place of service codes in the MarketScan® data, including primary care physician (PCP) visits, specialist visits, preventive care visits, outpatient visits for diagnostic services, ED visits, urgent care visits, hospital stays, and a broader measure that captured any clinician office visit (i.e., PCP, specialist, or preventive care service office visits). PCP and specialist visits consisted mainly of new and established patient office visits, while preventive care visits included well-patient visits, screening mammography, pap smear, immunizations, and colonoscopies. Outpatient diagnostic services included lab and imaging services. Service categories were not mutually exclusive, though overlap was minimal except for the broader office visit outcome. Appendix Table A2 describes the most common procedures and diagnoses for each service category.

For each category, we constructed a measure equal to 1 if the individual used the service in the calendar year and 0 otherwise (extensive margin use). We also created a measure of intensity using the count of the number of visits/stays conditional on at least one visit/stay in the calendar year (intensive margin use).

### Statistical analyses

We estimated a series of difference-in-differences (DD) models comparing workers in MSAs exposed to a PSL mandate to workers in unexposed MSAs. The key independent variable was an indicator for whether workers in an MSA had been exposed to a PSL mandate in a given year. Because mandate exposure among MSAs in our sample varied over time and we anticipated potential heterogenous treatment effects, we used an approach developed by Callaway and Sant’Anna (2021) to estimate our regression models (see Appendix Section A1 for a full description).^27^ Callaway and Sant’Anna (CS) estimates were derived from a DD model that restricted comparisons to those between exposed MSAs and MSAs that were never exposed to a PSL mandate during our sample period. These estimates represent separate group-time treatment effects, where a group is composed of all MSAs exposed to a PSL mandate each year. We aggregated these group-time treatment effects into a single weighted average summary measure. We incorporated the covariates described above into the CS estimation process by applying inverse probability of treatment weighting (IPTW), which reweights observations based on their probability of treatment. This approach, used within the CS framework, improves balance between treatment and control groups and can help to reduce potential confounding. Additionally, this method leverages a doubly-robust estimation strategy, which ensures consistent estimation of treatment effects as long as either the propensity score model or the outcome regression model is correctly specified. Standard errors were robust to heteroskedasticity and clustered at the state level to allow for within-state correlation.^28^ We reported estimates from this procedure as our “adjusted” estimates along with “unadjusted” estimates that were simple mean comparisons across exposed and unexposed MSAs before and after PSL mandate exposure. All analyses were performed using Stata version 18.

In addition to the CS method, we also estimated DD models using a two-way fixed effects (TWFE) approach. TWFE models included MSA fixed effects to control for time-invariant worker differences and year fixed effects to control for time-varying changes common to workers across all MSAs, along with the covariates described above. We used IPTW to improve baseline covariate balance between MSAs with and without PSL mandate exposure. This involved estimating a logistic regression model using the 2011 through 2014 baseline sample years, with mandate exposure as the dependent variable and the covariates described above. We excluded baseline outcome measures from the IPTW model to avoid issues with mean reversion.^29^ Using predicted probabilities of treatment from the logistic regression, we assigned propensity scores of 1 to exposed MSAs, while unexposed MSAs were assigned a propensity score equal to [(predicted probability of treatment/(1–predicted probability of treatment)] (i.e., the inverse probability of treatment selection). Appendix Section A2 provides further details on the propensity score matching technique. All TWFE models were weighted using the product of the IPTW weights and the number of sample individuals in an MSA and standard errors were clustered at the state level. Finally, we conducted a Goodman-Bacon decomposition for each outcome to assess potential bias in TWFE estimation due to differential treatment timing and treatment effect heterogeneity.^30^

The validity of our DD design requires that baseline differences in outcomes for MSAs exposed to a PSL mandate and unexposed MSAs remain stable over time in the absence of treatment (i.e., the “parallel trends assumption”). While not directly testable, we provided evidence of this by testing for differential trends in outcomes for exposed and unexposed MSAs in the periods before mandate adoption. These “event study” estimates also allowed us to trace out heterogenous effects of PSL mandates on health care utilization over time. Another potential threat to validity is the possibility of compositional changes in our sample that differ between exposed and unexposed MSAs. For instance, PSL mandates could be associated with changes in private insurance coverage, altering the characteristics of the populations being compared. While prior research has found little evidence linking PSL mandates to changes in health insurance coverage,^31^ we explored this possibility within our sample. Specifically, we replaced health care utilization outcomes in our regression models with demographic factors such as sex, age, race and ethnicity, and educational attainment at the MSA level. This approach allowed us to assess whether population characteristics among private sector workers with employer-sponsored health insurance changed differently over time between MSAs with and without PSL mandates.

### Sensitivity analyses

We leveraged the availability of employment industry in the MarketScan® data to conduct a sensitivity analysis that restricted our sample to workers in industries that had below-median PSL coverage rates during our sample period. We hypothesized that PSL mandates would have a larger effect on health care utilization for workers in these industries, as they would be more likely to gain coverage following mandate exposure. We estimated industry-specific PSL coverage rates using the National Health Interview Survey (NHIS), which asked whether paid sick leave was available at respondents’ current job.^32^ For comparability with our MarketScan® data, we restricted respondents to working adults ages 40 and 64 with employer-sponsored insurance coverage. This “low PSL sample” included workers in the agricultural, construction, retail, and service industries. We also estimated several alternative specifications for both samples, including models estimated at the individual-level rather than the MSA-level and models that replaced the MSA fixed effects with individual fixed effects.

### Role of the funding source

The funder had no role in study design, data collection, analysis, interpretation, manuscript preparation, review, or approval.

## Results

Table 1 reports baseline descriptive statistics for MSAs with and without PSL mandate exposure, both before and after applying the IPTW weighting procedure described above. Nearly half of sample individuals were female, and the average age in both exposed and unexposed MSAs was approximately 50 years. MSAs exposed to PSL mandates had a larger share of Hispanic residents and a smaller share of Non-Hispanic Black and Non-Hispanic white residents than unexposed MSAs. A larger share of residents in exposed MSAs were college-educated, and while state unemployment rates were higher in exposed MSAs, uninsured rates were lower. The IPTW procedure improved baseline covariate balance for nearly all characteristics listed in Table 1 as measured by changes in the standardized differences between exposed and unexposed MSAs.

**Table 1:** Baseline sample descriptive statistics, 2011–2014.

	Mandate	No mandate	Unweighted standardized difference	Weighted no mandate	Weighted standardized difference
*Clinician/service type outcomes*					
PCP Visit (%)	71.16	74.82	−0.566	74.71	−0.548
Number of PCP Visits	3.12	3.39	−0.619	2.99	0.310
Specialist visit (%)	42.22	44.46	−0.284	40.93	0.164
Number of specialist visits	3.03	3.38	−0.888	2.94	0.209
Preventive visit (%)	35.79	25.87	0.941	35.96	−0.016
Number of preventive visits	1.08	1.07	0.275	1.08	−0.139
Diagnostic visit (%)	29.37	30.63	−0.182	29.99	−0.090
Number of diagnostic visits	1.90	1.86	0.196	1.92	−0.091
*Clinical setting outcome*					
Office visit (%)	79.13	82.97	−0.563	82.33	−0.470
Number of office visits	4.72	5.11	−0.702	4.60	0.223
Emergency department visit (%)	9.85	11.57	−0.742	10.42	−0.243
Number of emergency department visits	1.28	1.30	−0.253	1.29	−0.168
Urgent care visit (%)	6.25	4.63	0.393	5.41	0.202
Number of urgent care visits	1.39	1.42	−0.151	1.37	0.054
Hospital stay (%)	3.58	4.08	−0.635	3.71	−0.158
Number of hospital stays	1.24	1.28	−0.378	1.24	0.011
*Sample characteristics*					
Female (%)	47.02	41.58	0.704	47.18	−0.021
Average age	51.38	51.14	0.316	51.34	0.051
*MSA characteristics (%)*					
Non-hispanic black	5.21	10.26	−0.647	5.05	0.021
Hispanic	14.24	8.32	0.401	16.46	−0.150
Other race or ethnicity	7.08	3.72	0.574	5.45	0.279
Non-hispanic white	73.47	77.70	−0.254	73.05	0.025
Less than high school	6.34	4.93	0.360	4.52	0.465
High school	31.77	36.24	−0.715	33.59	−0.292
Some college	26.65	25.30	0.360	27.53	−0.233
College	35.24	33.53	0.205	34.35	0.106
*State characteristics (%)*					
State unemployment rate	7.77	5.97	1.028	7.29	0.276
Uninsurance rate	15.04	16.12	−0.169	14.02	0.161
MSA-years	248	740			

*Notes*: The baseline period includes years 2011 through 2014 and the unit of observation is the MSA-by-state. We used an inverse probability of treatment weighting propensity score matching procedure to improve covariate balance on baseline covariates. The standardized difference is calculated according to Austin (2015).^33^ The means for the outcome variables in Tables 2 and 3 differ from those in Table 1 because they are weighted differently. In Tables 2 and 3, means are weighted by the product of the inverse probability of treatment weights and the number of sample individuals in an MSA. In Table 1, means are weighted only by the inverse probability of treatment weights.

Tables 2 and 3 include our DD estimates of the impact of PSL mandates on utilization by clinician/service type and clinical setting. Columns 1 through 4 contain unadjusted mean values for each outcome by PSL mandate exposure. Column 5 contains unadjusted estimates, which are the mean post-to-pre differences for exposed MSAs minus the mean post-to-pre differences for unexposed MSAs. Column 6 includes our adjusted CS estimates along with standard errors and 95% confidence intervals. Results from our Goodman-Bacon decompositions indicated that TWFE estimates, which included both MSAs that never adopted PSL mandates during our sample period and those that did adopt prior to their adoption as controls, were biased toward the null for several of the outcomes we studied (see Appendix Tables A3 and A4). As such, we focused our description of results on the estimates derived using the CS approach that is designed to eliminate this bias. Estimates from the TWFE models are reported in Appendix Tables A7 and A8.

**Table 2:** Estimates of the impact of PSL mandates on health care utilization by clinician/service type, 2011–2019.

Outcome	Exposed MSAs		Unexposed MSAs		Unadjusted DD estimate	Adjusted DD estimate (standard error) [95% confidence interval]
	Pre-mandate	Post-mandate	Pre-mandate	Post-mandate		
	(1)	(2)	(3)	(4)	(5)	(6)
PCP visit (%)	72.12	74.40	75.09	75.32	2.05	4.79∗∗∗ (1.74) [1.39, 8.19]
Number of PCP visits	3.05	2.97	3.19	3.01	0.104	−0.001 (0.046) [−0.091, 0.089]
Specialist visit (%)	41.57	46.22	44.59	48.20	1.04	2.71∗∗∗ (0.882) [0.980, 4.44]
Number of specialist visits	3.08	3.23	3.19	3.27	0.065	0.171∗∗∗ (0.058) [0.057, 0.285]
Preventive visit (%)	36.93	42.59	38.34	46.28	−2.29	2.75∗ (1.59) [−0.358, 5.86]
Number of preventive visits	1.09	1.11	1.11	1.13	−0.012	0.028∗ (0.015) [−0.001, 0.057]
Diagnostic visit (%)	30.79	33.48	35.04	35.66	2.06	2.20∗∗∗ (0.505) [1.21, 3.19]
Number of diagnostic visits	1.87	2.02	1.91	2.02	0.033	0.078∗∗ (0.036) [0.008, 0.148]

*Notes*: Unadjusted DD estimates were calculated as the outcome difference between the post- and pre-mandate periods for the unexposed MSAs subtracted from the difference between the post- and pre-mandate periods for the exposed MSAs. Adjusted DD estimates were derived from the Callaway and Sant’Anna procedure for staggered intervention timing and use stabilized inverse probability weighting for improved covariate balance. The unit of observation is the MSA-by-year, standard errors clustered at the state level, and all models are weighted by the number of sample individuals residing in each MSA multiplied by the inverse probability of treatment weights. The sample consisted of 247 MSAs, 62 of which were exposed to a PSL mandate, observed over a 9-year period for a total of 2223 observations.

p < 0.10∗, p < 0.05∗∗, p < 0.01∗∗∗.

**Table 3:** Estimates of the impact of PSL mandates on health care utilization by clinical setting, 2011–2019.

Outcome	Exposed MSAs		Unexposed MSAs		Unadjusted DD estimate	Adjusted DD estimate (standard error) [95% confidence interval]
	Pre-mandate	Post-mandate	Pre-mandate	Post-mandate		
	(1)	(2)	(3)	(4)	(5)	(6)
Office visit (%)	79.05	82.16	82.60	84.35	1.36	4.63∗∗∗ (1.66) [1.40, 7.88]
Number of office visits	4.57	4.76	4.93	5.01	0.110	0.031 (0.069) [−0.104, 0.166]
Emergency department visit (%)	9.75	9.83	11.51	11.70	−0.110	0.516 (0.316) [−0.103, 1.14]
Number of emergency department visits	1.26	1.26	1.31	1.32	−0.007	0.065 (0.040) [−0.014, 0.143]
Urgent care visit (%)	6.15	8.22	6.90	11.62	−2.66	−1.79 (1.22) [−4.19, 0.600]
Number of urgent care visits	1.40	1.41	1.40	1.48	−0.067	0.102∗∗ (0.042) [0.019, 0.184]
Hospital stay (%)	3.58	3.38	4.07	3.77	0.100	0.346∗∗ (0.162) [0.028, 0.664]
Number of hospital stays	1.25	1.28	1.26	1.29	−0.000	0.033 (0.030) [−0.027, 0.092]

*Notes*: Unadjusted DD estimates were calculated as the outcome difference between the post- and pre-mandate periods for the unexposed MSAs subtracted from the difference between the post- and pre-mandate periods for the exposed MSAs. Adjusted DD estimates were derived from the Callaway and Sant’Anna procedure for staggered intervention timing and use stabilized inverse probability weighting for improved covariate balance. The unit of observation is the MSA-by-year, standard errors clustered at the state level, and all models are weighted by the number of sample individuals residing in each MSA multiplied by the inverse probability of treatment weights. The sample consisted of 247 MSAs, 62 of which were exposed to a PSL mandate, observed over a 9-year period for a total of 2223 observations.

p < 0.10∗, p < 0.05∗∗, p < 0.01∗∗∗.

**Fig. 1:**
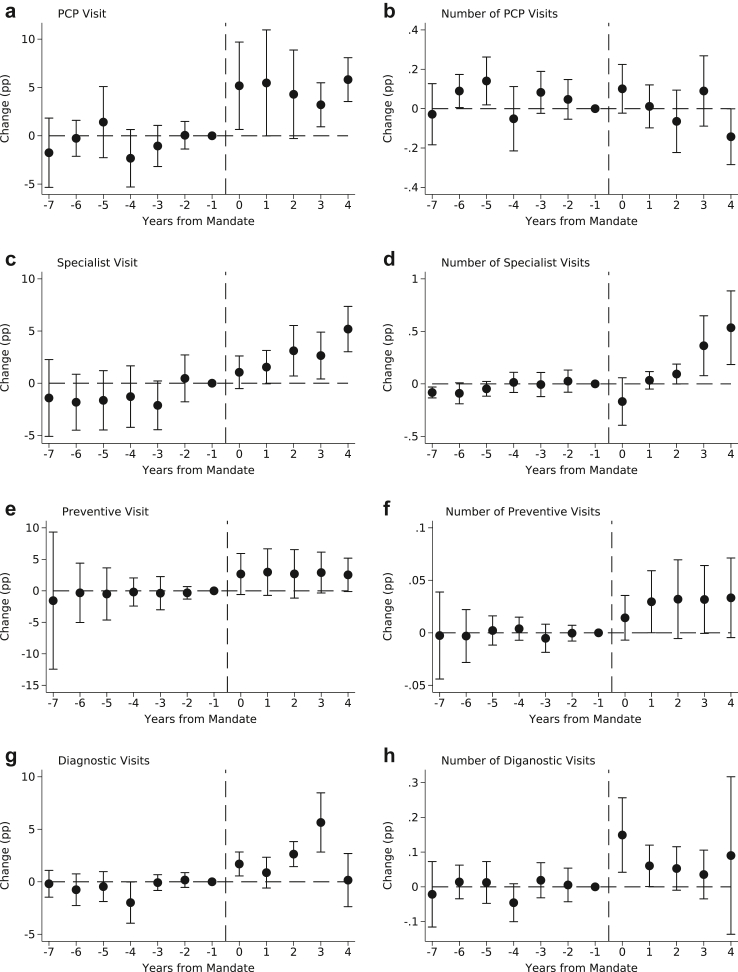
Event Study Estimates of the Impact of PSL Mandates on Health Care Utilization by Clinician/Service Type. *Notes*: Event study estimates were calculated using the Callaway and Sant’Anna procedure for staggered intervention timing using long gaps for comparability to traditional TWFE event studies. Models employed stabilized inverse probability weighting for improved covariate balance using MSA-by-state-by-year race and ethnicity composition, educational attainment, state-level share uninsured, and state-level unemployment rates. The vertical dashed line indicates the timing of PSL mandate exposure. The unit of observation is the MSA-by-state-by-year and standard errors clustered at the state level.

Estimates in Table 2 show that workers in MSAs that were exposed to a PSL mandate experienced a 4.79pp (95% CI, 1.39–8.19) increase in the probability of a PCP visit, representing a 6.64% increase from a baseline mean of 72.12%, compared to those in unexposed MSAs. PSL mandates were also associated with a 2.71pp (6.52%; 95% CI, 0.980–4.44) increase in the probability of a specialist visit, a 2.75pp (7.45%; 95% CI, −0.358 to 5.86) increase in the probability of a preventive care visit, and a 2.20pp (7.15%; 95% CI, 1.21–3.19) increase in the probability of an outpatient diagnostic visit. PSL mandates were associated with an increase in the annual number of specialist visits (0.171pp; 5.55%; 95% CI, 0.057–0.285), preventive visits (0.028pp; 2.57%; 95% CI, −0.001 to 0.057), and outpatient diagnostic visits (0.078pp, 4.17%; 95% CI, 0.008–0.148). Event study estimates for the outcomes in Table 2 are presented in Fig. 1 and indicate no evidence of diverging pre-period trends, which supports the validity of the DD research design (see Appendix Figure A5 for TWFE event study estimates). However, event study estimates for outpatient diagnostic visits were unstable in the post-period and so results for this outcome should be interpreted with caution.

**Fig. 2:**
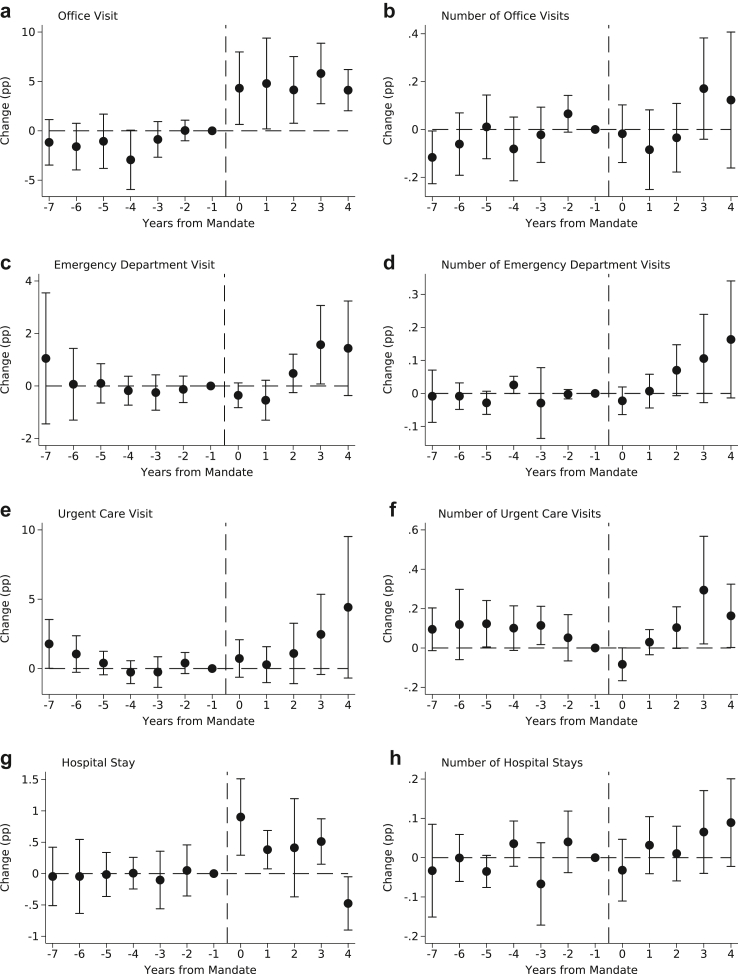
Event Study Estimates of the Impact of PSL Mandates on Health Care Utilization by Clinical Setting. *Notes*: Event study estimates were calculated using the Callaway and Sant’Anna procedure for staggered intervention timing using long gaps for comparability to traditional TWFE event studies. Models employed stabilized inverse probability weighting for improved covariate balance using MSA-by-state-by-year race and ethnicity composition, educational attainment, state-level share uninsured, and state-level unemployment rates. The vertical dashed line indicates the timing of PSL mandate exposure. The unit of observation is the MSA-by-state-by-year and standard errors clustered at the state level.

Table 3 includes estimates of the impact of PSL mandates on: office visits (PCP, specialist, or preventive service office visits), ED visits, urgent care visits, and hospital stays. PSL mandates were associated with a 4.63pp (5.86%; 95% CI, 1.40–7.88) increase in the probability of an office visit and a 0.346pp (9.66%; 95% CI, 0.028–0.664) increase in hospital stays. While the estimated effect for hospital stays is large, event study estimates in Fig. 2 show no clear pattern in the post-mandate period and indicate no difference in hospital stays for those living in exposed MSAs and unexposed MSAs four years after PSL mandate adoption (see Appendix Figure A6 for TWFE event study estimates). We found no statistically significant association between PSL mandates and the probability of an ED visit or an urgent care visit. PSL mandates were not associated with changes in intensive margin measures of service use in Table 3, except for the annual number of urgent care visits, which increased by 0.102pp (7.29%; 95% CI, 0.019–0.184) following PSL mandate exposure.

Appendix Tables A9 and A10 report results for the sensitivity analysis that restricted the sample to workers in industries with low rates of PSL coverage. Consistent with our hypothesis, estimates of the association between PSL mandates and health care utilization were typically larger in relative magnitude for those working in low PSL industries. For example, workers in exposed MSAs employed in low PSL industries experienced a 9.34% increase in the probability of a PCP visit compared to a 6.64% increase for the full sample. Relative magnitudes also were larger among workers in low PSL industries for specialist (7.93% vs. 6.52%), preventive care (13.81% vs. 7.45%), outpatient diagnostic (7.87% vs. 7.15%), and office (9.59% vs. 5.86%) visits.

Appendix Tables A11 through A14 present results from models using individual-level data and individual fixed effects, which rely on variation among individuals with differing exposure to PSL mandates, rather than the variation across MSAs used in our baseline models. The results, which show increased PCP, specialist, outpatient diagnostic, and office visits associated with PSL mandates—especially among workers in low PSL industries—align with our main findings in Tables 2 and 3 Although precision often declined in models with individual fixed effects due to limited identifying variation, the consistency of point estimates across specifications supports the robustness of our baseline results.

Finally, Appendix Table A15 presents estimates of demographic changes in MSAs exposed to PSL mandates compared to unexposed MSAs following mandate exposure. Changes were small in magnitude and generally not statistically significant at traditional levels, indicating minimal shifts in the composition of private sector workers with employer-sponsored health insurance associated with PSL mandates.

## Discussion

We estimated the association between PSL mandates, which require employers to provide paid time off for illness to qualified employees, and health care utilization for U.S. workers between 2011 and 2019. We found that PSL mandates were positively associated with extensive margin measures of health service utilization, including the likelihood that a worker visited their PCP or a specialist and experienced a preventive care or outpatient diagnostic visit in the past year. Among those with at least one clinical encounter, we found mixed evidence for the relationship between PSL mandates and encounter frequency. PSL mandates were not associated with changes in the annual number of PCP visits, office visits, ED visits, or hospital stays, but were associated with an increase in the annual number of specialist visits, preventive care visits, outpatient diagnostic visits, and urgent care visits. Estimates were generally larger for workers who were employed in industries that historically offered low rates of PSL coverage.

We interpret this pattern of findings as suggestive evidence that gaining PSL coverage can reduce barriers to care and increase care-seeking among those who may otherwise forego clinical visits and preventive care services. The weaker relationship between PSL mandates and conditional measures of visit frequency may indicate that PSL coverage primarily encourages individuals to seek care when they otherwise would not, rather than altering the frequency of visits for those already engaging in regular care.

In general, the magnitudes of our estimates were modest, though many of the workers in our sample presumably had PSL coverage prior to the adoption of a mandate. Estimates suggest that, on average, 28% of private sector workers gain PSL coverage as a result of exposure to a PSL mandate.^6^ Scaling our results by this estimated coverage gain suggests that mandates were associated with a (4.63/0.28)/79.05 = 20.92% increase in the probability of a clinician office visit (PCP, specialist, or preventive care office visit), a (4.79/0.28)/72.12 = 23.72% increase in the probability of a PCP visit, a (2.71/0.28)/41.57 = 23.28% increase in the probability of a specialist visit, and a (2.75/0.28)/39.93 = 24.60% increase in the probability of a preventive care visit. However, this scaling exercise should be interpreted with caution, as it relies on the assumption that changes in health care utilization following mandate exposure are driven entirely by those gaining PSL coverage.

Our findings are directionally consistent with those from prior studies examining PSL and health care utilization. However, our estimated associations tend to be smaller compared to studies using adjusted comparisons between those with and without PSL, likely because our methodology is better able to address confounding from selection into coverage. For example, in a recent review of the literature, Lamsal et al. (2021) reported that PSL coverage was associated with a 33% higher likelihood of having a past year doctor visit,^34^ whereas our scaled estimate is approximately two-thirds of this size. Alternatively, the magnitude of our estimates are similar to smaller-scale studies that, like ours, attempt to address selection bias by using PSL mandates as natural experiments that generate exogenous variation in coverage, but rely on data from a single state or examine a smaller range of outcomes.^10^^,^^21^

### Limitations

Though administrative claims data minimize misreporting and recall bias,^35^ our data were restricted to claims for privately insured workers aged 40–64 years. While this likely captures a sizable share of clinical encounters among those gaining PSL coverage, it does not provide insights for younger workers or those who are not privately insured. Our results are also not generalizable to people who are not employed and may only indirectly benefit from PSL mandates, such as children and retirees.^36^, ^37^, ^38^ Additionally, because we restricted our sample to workers with continuous health plan coverage to ensure complete capture of health service use, our findings may not generalize to those experiencing gaps in health insurance coverage. Finally, as our analysis was limited to workers residing in MSAs, our findings primarily reflect PSL's effects on health care utilization for those living in urban areas.

One key assumption of our research strategy is that no unobserved confounders changed differentially between treatment and control units at the time of PSL mandate adoption and that no other policies were implemented concurrently in ways that systematically bias our estimates. While we cannot entirely rule out this possibility, we found no evidence that concurrent policy adoption was common among municipalities in our sample or that trends in observed covariates between treatment and control groups changed meaningfully at the time of mandate exposure (see Appendix Table A15).

Dichotomizing mandate exposure is a requirement of the analytic procedure used in our analysis, but results in misclassification for mandates effective mid-year. The practical implication of this misclassification is to attenuate our estimated association between PSL mandates and health care utilization. Another limitation is our reliance on worker residence as opposed to worker place of employment to identify PSL mandate exposure. This misclassification likely attenuates our estimates in cases of sub-state mandates, where it is more common for workers to commute from areas with no mandate to areas with mandate exposure.^39^

### Conclusion

Studies examining the association between PSL and health care utilization often rely on analytical methods prone to selection bias or focus on narrow policy settings. In contrast, we conducted a large-scale study of the association between PSL coverage and health care utilization, including PCP visits, specialist visits, encounters for preventive care and diagnostic services, clinician office visits, ED and urgent care visits, and hospital stays, for private sector U.S. workers using a plausibly exogenous source of variation in PSL exposure. Our findings lend support to a growing evidence base that has found workplace flexibilities, like PSL, increase access to health care services.

## Contributors

KC, MFP, SP, and JAS contributed equally to study conceptualisation, formal analysis, funding acquisition, methodology, and writing-review & editing. KC had access to raw data, was responsible for data curation, and writing-original draft. SP had access to raw data and contributed to data curation.

## Data sharing statement

The data we use in this study is proprietary and we do not have permission to share the data. However, we will make our code available so that those with access to the data can replicate our findings. Please email Kevin Callison (kcallison@tulane.edu) to request a copy of the code.

## Declaration of interests

All authors reported receiving grant funding from the National Cancer Institute. KC received funding from the American Cancer Society, the Commonwealth Fund, the Eunice Kennedy Shriver National Institute of Child Health and Human Development, Flatiron Health, the W.K. Kellogg Foundation, and the Louisiana Department of Health. MFP received funding from the American Cancer Society, Flatiron Health, and the National Institute of Mental Health. SP received funding from the American Cancer Society and Flatiron Health. JAS is a member of the Data Monitoring Committee of the Medullary Thyroid Cancer Consortium Registry supported by Novo Nordisk, Astra Zeneca, and Eli Lilly, and institutional research funding was received from Exelixis and Eli Lilly. JAS also received funding from the American Cancer Society, Flatiron Health, and the National Institute on Minority Health and Health Disparities. No other disclosures were reported.
