# Supplementary material for: Health care utilization following the adoption of U.S. paid sick leave mandates: a cohort study using health insurance claims data
Source: Lancet Reg Health Am. 2025 Jul 7;49:101174. doi: 10.1016/j.lana.2025.101174 (PMC12273583; doi:10.1016/j.lana.2025.101174)
Supplement: Appendix Tables [file mmc1.pdf]

Appendix

Section A1. Difference-in-differences Estimation Method

Section A2. Propensity Score Matching Technique

Table A1. Paid Sick Leave Mandates, 2015-2019

Table A2. Most Frequent Current Procedural Terminology or Diagnosis Codes by Service Category

Table A3. Goodman-Bacon Decompositions for Clinician/Service Type Outcomes

Table A4. Goodman-Bacon Decompositions for Clinical Setting Outcomes

Figure A5. Two-Way Fixed Effect Event Study Estimates of the Impact of PSL Mandates on Health Care Utilization by Clinician/Service Type

Figure A6. Two-Way Fixed Effect Event Study Estimates of the Impact of PSL Mandates on Health Care Utilization by Clinical Setting

Table A7. Two-Way Fixed Effect Estimates of the Impact of PSL Mandates on Health Care Utilization by Clinician/Service Type, 2011 – 2019

Table A8. Two-Way Fixed Effect Estimates of the Impact of PSL Mandates on Health Care Utilization by Clinical Setting, 2011 – 2019

Table A9. Estimates of the Impact of PSL Mandates on Health Care Utilization by Clinician/Service Type – Low PSL Sample, 2011 – 2019

Table A10. Estimates of the Impact of PSL Mandates on Health Care Utilization by Clinical Setting – Low PSL Sample, 2011 – 2019

Table A11. Individual-Level Callaway and Sant'Anna Estimates of the Impact of PSL Mandates on Health Care Utilization by Clinician/Service Type, 2011 – 2019

Table A12. Individual-Level Callaway and Sant'Anna Estimates of the Impact of PSL Mandates on Health Care Utilization by Clinical Setting, 2011 – 2019

Table A13. Individual-Level Two-Way Fixed Effect Estimates of the Impact of PSL Mandates on Health Care Utilization by Clinician/Service Type, 2011 – 2019

Table A14. Individual-Level Two-Way Fixed Effect Estimates of the Impact of PSL Mandates on Health Care Utilization by Clinical Setting, 2011 – 2019

Table A15. Demographic Characteristic Changes Associated with PSL Mandates

Table A16. Strobe Statement

Appendix References

Section A1: Difference-in-differences Estimation Method

We used a difference-in-differences (DD) regression design to estimate the association between PSL mandates and health care utilization. DD models compare changes for units exposed to a treatment or intervention (PSL mandates in our case) to unexposed units before and after exposure. Our DD model was specified as follows:

$$(1)\ Y_{mt} = \alpha + \gamma PSL_{mt} + \beta X_{mt} + \delta_m + \tau_t + \varepsilon_{mt}$$

where Y_{mt} represents either the probability of a clinical encounter or the conditional number of clinical encounters in MSA m in year t. PSL_{mt} is a variable that captures PSL mandate exposure and is equal to 1 in the year that an MSA becomes exposed to a PSL mandate and in the following years and is equal to 0 for unexposed MSAs. X_{mt} is a vector of MSA-by-state-by-year characteristics that included age, sex, race and ethnicity composition, educational attainment, share uninsured, and state-level unemployment rates and share of the population living in poverty. Finally, τ_t and δ_m represent indicator variables for each year and each MSA-by-state combination, respectively. Standard errors are represented by ε_{mt} and were clustered at the state level.

DD models of this type are often estimated using a two-way fixed effects (TWFE) procedure. However, recent work has highlighted the potential for TWFE estimation of DD models with staggered treatment timing and heterogenous treatment effects to produce biased coefficient estimates.[1-3] This bias can arise because TWFE estimation compares exposed to unexposed units, but when treatment exposure varies over time, also compares units exposed earlier in the sample period to units exposed later in the sample period and vice versa. The TWFE DD coefficient estimate is a weighted sum of these two-way comparisons.[1] When treatment effects vary over time, the late-to-early comparisons become problematic because outcomes for the units exposed earlier in the sample period, including the time-varying treatment effects, are subtracted from outcomes for the units exposed later in the sample period.

To address this bias, we estimated our DD models using a procedure developed in Callaway and Sant'Anna (2021) that restricts comparisons to those between exposed and unexposed units and calculated separate group-time treatment effects where each group is comprised of MSAs exposed to a PSL mandate in the same calendar year.[4] The Callaway and Sant'Anna estimator that we used took the following form:

$$(2)\ ATT_{g,t} = \frac{1}{n}\sum_{m=1}^{n} \frac{\mathbf{1}\{G_m = g\}}{\mathbb{E}(G_m = g)} \left(Y_{m,t} - Y_{m,g-1} - \mathbb{E}[Y_{m,t} - Y_{m,g-1} | X, C_m = 1]\right)$$

where $ATT_{g,t}$ is the average treatment effect for the treated units (i.e., MSAs exposed to a PSL mandate) at time t that were treated in period g, with period g corresponding to a year between 2015 and 2018 in our sample. G_m indicates that unit m is a member of group G, whose members were all exposed to treatment in period g. In our case, we have four groups (G) comprised of MSAs exposed to PSL mandates in 2015, those exposed in 2016, those exposed in 2017, and those exposed in 2018. C_m is equal to 1 for units that were never exposed to treatment and Y and X are as defined previously. Equation (2) shows that the Callaway and Sant'Anna procedure produces treatment effect estimates for each group, G, at each time, t, that are derived from a

process that compares outcomes for group G member units to outcomes for never-treated units at time t relative to the period immediately preceding group G's treatment period, $g - 1$.

Finally, as this process generates an average treatment effect for each group-time combination (32 separate estimates in our case), we aggregated these individual average treatment effect estimates into a single parameter that is comparable to a typical TWFE DD estimate. To do so, we calculated the following:

$$(3)\ ATT_{aggregate} = \sum_{g \in \mathcal{G}} \left(\frac{1}{\mathcal{T} - g + 1} \sum_{t=g}^{\mathcal{T}} ATT_{g,t} \right) P(G = g | g \leq t)$$

where $\mathcal{G}$ represents the support of G and excludes units that were never exposed to treatment and $\mathcal{T}$ represents the number of sample data periods (i.e., 8 years in our sample). The intuition behind equation (3) is that we calculated the mean treatment effect ($ATT_{g,t}$) for each exposed group ($G = g$), over their post-exposure period ($g \leq t$) and then averaged those means across all exposed groups to arrive at a single DD parameter estimate.

Section A2: Propensity Score Matching Technique

We used an inverse probability of treatment weighting propensity score matching procedure to achieve covariate balance on baseline covariates. We did not include baseline outcome measures in the matching procedure to avoid issues with mean reversion.[5] This technique consisted of a two-step procedure, where the first step was to estimate a logistic regression model using the 2012 through 2014 baseline years with an indicator for mandate exposure as the dependent variable and age, sex, MSA-by-state-by-year race and ethnicity composition, educational attainment, and state-level share uninsured and unemployment rates as independent variables. The second step involved using the predicted probability of treatment from the logistic regression to assign propensity scores to exposed and unexposed MSAs. Exposed MSAs were assigned a propensity score of 1, while unexposed MSAs were assigned a propensity score equal to [(predicted probability of treatment / (1 - predicted probability of treatment)] (i.e., the inverse probability of treatment selection). We then used the product of the propensity scores and the number of sample individuals in each MSA-state as weights to calculate descriptive statistics and as weights in our regression models.[6,7]

Table A1: Paid Sick Leave Mandates, 2015-2019

Mandate Jurisdiction	Year	Minimum Firm Size (# employees)	Accrual Rate/Maximum	Qualified Private Sector Workers	Affected MSAs
California	2015	No Minimum	1 hour per 30 hours worked / 48 hours	Work for the same employer for at least 30 days within a year and satisfy a 90-day probationary period.	Bakersfield, CA; Chico, CA; El Centro, CA; Fresno, CA; Hanford-Corcoran, CA; Los Angeles-Long Beach-Anaheim, CA; Madera, CA; Merced, CA; Modesto, CA; Napa, CA; Oxnard-Thousand Oaks-Ventura, CA; Redding, CA; Riverside-San Bernardino-Ontario, CA; Sacramento-Roseville-Folsom, CA; Salinas, CA; San Diego-Chula Vista-Carlsbad, CA; San Jose-Sunnyvale-Santa Clara, CA; San Luis Obispo-Paso Robles, CA; Santa Cruz-Watsonville, CA; Santa Maria-Santa Barbara, CA; Santa Rosa-Petaluma, CA; Stockton, CA; Vallejo, CA; Visalia, CA; Yuba City, CA
Massachusetts	2015	11+	1 hour per 30 hours worked / 40 hours	All workers	Barnstable Town, MA; Boston-Cambridge-Newton, MA-NH; Pittsfield, MA; Springfield, MA; Worcester, MA-CT
Philadelphia, PA	2015	10+	1 hour per 40 hours worked / 40 hours	Must work at least 40 hours a year in Philadelphia. Excludes independent contractors, seasonal workers, adjunct professors, employees hired for less than 6 months, interns, pool employees, employees covered by collective bargaining agreements.	Philadelphia-Camden-Wilmington, PA-NJ-DE-MD

Trenton, NJ	2015	No minimum	1 hour per 30 hours worked / 40 hours if firm size is 10+, 24 hours otherwise	Must work more than 80 hours in a calendar year in the city of Trenton. Excludes members of a construction union who are enrolled or have graduated from a registered apprenticeship program and are covered by a collective bargaining agreement.	Trenton-Princeton, NJ
Oregon	2016	10+ (6+ if located in a city with 500k residents)	1 hour per 30 hours worked / 40 hours	All workers	Bend, OR; Eugene-Springfield, OR; Medford, OR
Arizona	2017	No minimum	1 hour per 30 hours worked / 40 hours if firm size is 15+, 24 hours otherwise	All workers	Flagstaff, AZ; Lake Havasu City-Kingman, AZ; Phoenix-Mesa-Chandler, AZ; Prescott, AZ; Tucson, AZ; Yuma, AZ
Chicago & Cook County, IL	2017	No minimum	1 hour per 40 hours worked / 40 hours	Must work at least 80 hours for an employer in Chicago/Cook County within any 120-day period.	Chicago-Naperville-Elgin, IL-IN-WI
Minneapolis, MN & St. Paul MN	2017	5+ (Minneapolis only)	1 hour per 30 hours worked / 48 hours	All workers	Minneapolis-St. Paul-Bloomington, MN-WI
Spokane, WA	2017	No minimum	1 hour per 30 hours worked / 24 hours	Must perform more than 240 hours of work physically in the city of Spokane in a calendar year. Excludes seasonal and domestic workers, independent contractors, work study students, and those employed by firms engaged in construction work.	Spokane-Spokane Valley, WA
Vermont	2017	No minimum	1 hour per 52 hours worked / 24 hours in	Must be 18 or older, work an average of 18 or more hours per week during the year, and	Burlington-South Burlington, VT

			2017/2018, 40 hours after 2018	work more than 20 weeks in a 12-month period. Excludes bona fide independent contractors.	
Maryland	2018	15+	1 hour per 30 hours worked / 40 hours	Excludes employees working fewer than 12 hours a week, independent contractors, real estate salespersons, those under the age of 18, those employed in the agricultural sector, construction workers covered by collective bargaining, temporary workers, and commission-only workers.	Baltimore-Columbia-Towson, MD; Salisbury, MD-DE
Rhode Island	2018	18+	1 hour per 35 hours worked / 24 hours in 2018, 32 hours in 2019, and 40 hours after 2019	Must perform more work in Rhode Island than in any other state during the past 12 months. Excludes per diem nurses working at health care facilities who are under no obligation to work a regular schedule and receives higher pay than others who work a regular schedule while performing the same job.	Providence-Warwick, RI-MA
New Jersey	2018	No minimum	1 hour per 30 hours worked / 40 hours	Excludes those employed in the construction industry under a union contract, per diem health care employees, and independent contractors.	Atlantic City-Hammonton, NJ; Ocean City, NJ
Washington	2018	No minimum	1 hour per 40 hours worked / No maximum	Excludes individuals employed in hand harvest labor and paid on a piece rate basis, who commutes daily from their permanent	Bellingham, WA; Bremerton-Silverdale-Port Orchard, WA; Olympia-Lacey-Tumwater, WA; Yakima, WA

		residence to a farm, who had been employed in agriculture less than 13 weeks during the preceding calendar year. Individuals employed in casual labor in or about a private home. Individuals employed in a bona fide executive, administrative, or professional capacity. Newspaper vendors, carriers, or delivery people. Individuals whose duties require that they sleep at their place of employment. Vessel operating crews or individuals employed as seaman on a vessel other than an American vessel.	

Source: National Partnership for Women & Families Paid Sick Day Statutes: https://www.nationalpartnership.org/our-work/resources/economic-justice/paid-sick-days/paid-sick-days-statutes.pdf.

Notes: All mandates include coverage for eligible employees' and family members' care and allow for preventive care use. We dropped the following MSAs that were exposed to mandate before 2015 since these do not contribute identifying variation in our model: Bridgeport-Stamford-Norwalk, CT; Hartford-East Hartford-Middletown, CT; New Haven-Milford, CT; New York-Newark-Jersey City, NY-NJ-PA; Norwich-New London, CT; Portland-Vancouver-Hillsboro, OR-WA; San Francisco-Oakland-Berkeley, CA; Seattle-Tacoma-Bellevue, WA; and Washington-Arlington-Alexandria, DC-VA-MD-WV (includes the 2016 Montgomery County, MD mandate). Austin, TX adopted a PSL mandate in 2018, but that mandate has not yet been implemented due to a court order. In 2018, Michigan voters approved a ballot initiative that would have granted PSL coverage to most workers in the state, however the state legislature amended the ordinance to substantially limit its scope.

Table A2: Most Frequent Current Procedural Terminology or Diagnosis Codes by Service Category

Service Category/Code	Cumulative Frequency	Description
A. PCP Office Visits (CPT/HCPCS Codes)		
99213	0.373	Established patient office visit, 20-29 minutes
99214	0.677	Established patient office visit, 30-39 minutes
99396	0.835	Established well-patient visit for a patient aged 40 to 64
99212	0.867	Established patient office visit, 10-19 minutes
99203	0.895	New patient office visit, 30-44 minutes
99215	0.918	Established patient E&M office visit, 40-54 minutes
99204	0.935	New patient office visit, 45-59 minutes
99386	0.951	New patient preventive medicine services
99211	0.964	Established patient Office or Other Outpatient Services
99202	0.974	New patient E&M office visit, 15-29 minutes
B. Specialist Visits (CPT/HCPCS Codes)		
99213	0.376	Established patient office visit, 20-29 minutes
99214	0.650	Established patient office visit, 30-39 minutes
99203	0.756	New patient office visit, 30-44 minutes
99212	0.838	Established patient office visit, 10-19 minutes
99204	0.894	New patient office visit, 45-59 minutes
99215	0.927	Established patient E&M office visit, 40-54 minutes
99202	0.959	New patient E&M office visit, 15-29 minutes
99211	0.973	Established patient Office or Other Outpatient Services
99205	0.986	New patient E&M office visit, 60-74 minutes
99201	0.990	New patient E&M office visit, <10 minutes
C. Preventive Care Visits (CPT/HCPCS Codes)		
99396	0.199	Established well-patient visit for a patient aged 40 to 64
G0202	0.311	Screening mammography, bilateral including computer aided detection
77052	0.418	Digital mammogram with computer aided detection
90471	0.519	Immunization for Vaccines/Toxoids
88175	0.570	Cytopathology, cervical or vaginal
90658	0.599	Influenza Immunization
77067	0.626	Screening mammography, bilateral
45380	0.651	Colonoscopy, flexible; with biopsy, single or multiple
90715	0.676	Tetanus, diphtheria toxoids, and acellular pertussis vaccine
77063	0.698	Screening digital breast tomosynthesis, bilateral
D. Diagnostic Visits (CPT/HCPCS Codes)		
80061	0.064	Lipid panel
80053	0.118	Comprehensive metabolic panel
85025	0.168	Blood count; complete (CBC), automated (Hgb, Hct, RBC, WBC, and platelet count) and automated differential WBC count
83036	0.201	Hemoglobin; glycosylated (A1C)

84443	0.226	Thyroid stimulating hormone (TSH) testing
80050	0.247	Metabolic panel, complete blood count and thyroid stimulating hormone (TSH) level
82306	0.266	Vitamin D; 25 Hydroxy
93000	0.284	Routine electrocardiogram (ECG) with at least 12 leads, including tracing, interpretation, and report
84153	0.302	Prostate specific antigen (PSA) testing
80048	0.319	Basic metabolic panel without Glomerular Filtration Rate
E. ED Visits (ICD-9/10 Codes)		
78650	0.032	Chest pain, unspecified
78659	0.054	Other chest pain
R0789	0.073	Other chest pain
R079	0.088	Chest pain, unspecified
7840	0.102	Headache
78909	0.116	Abdominal pain, other specified site
7804	0.128	Dizziness and giddiness
7802	0.139	Transient alteration of awareness
78900	0.149	Abdominal pain, unspecified site
5921	0.158	Calculus of ureter
F. Urgent Care (ICD-9/10 Codes)		
4660	0.031	Acute bronchitis
4619	0.061	Acute sinusitis, unspecified
4659	0.087	Acute upper respiratory infections of unspecified site
J069	0.110	Acute upper respiratory infection, unspecified
462	0.132	Acute pharyngitis
J209	0.152	Acute bronchitis, unspecified
J0190	0.171	Acute sinusitis, unspecified
5990	0.189	Urinary tract infection, site not specified
J029	0.204	Acute pharyngitis, unspecified
R05	0.217	Cough
G. Hospital Stay (ICD-9/10 Codes)		
71536	0.025	Osteoarthrosis, localized, not specified whether primary or secondary, lower leg
27801	0.039	Morbid obesity
71535	0.052	Osteoarthrosis, localized, not specified whether primary or secondary, pelvic region and thigh
41401	0.065	Coronary atherosclerosis of native coronary artery
A419	0.078	Sepsis, unspecified organism
56211	0.090	Diverticulitis of colon (without mention of hemorrhage)
0389	0.102	Unspecified septicemia
42731	0.113	Atrial fibrillation
41071	0.124	Subendocardial infarction, initial episode of care
M1711	0.133	Unilateral primary osteoarthritis, right knee

Notes: PCPs and specialists were defined using service sub-category codes provided in the MarketScan® data. PCP service sub-category codes corresponded to clinicians categorized as “physician non-specialty" and specialist service sub-category codes corresponded to clinicians categorized as “physician specialty”. Preventive service visits were

defined using procedure group codes provided in the MarketScan® data that corresponded to preventive services (diagnostic mammography was excluded). Diagnostic services were defined using procedure group codes provided in the MarketScan® data that corresponded to the following list of services: bronchoscopy, laryngoscopy, upper GI endoscopy, cystourethroscopy, colposcopy, laparoscopy, hysteroscopy, psychiatric diagnostic services, ophthalmic diagnostic services, ENT diagnostic services, EKG, echocardiogram, cardiac catheterization, diagnostic radiology – other vascular, non-invasive peripheral vascular studies, spirometry, pulmonary function tests, allergy testing, nerve conduction tests/EMG, other neurology diagnostic services, x-ray, CT scan, PET scan, MRI, myelograms/discograms, aortograms, angiograms, lymphangiograms, venograms, diagnostic radiology, diagnostic ultrasound, echocardiogram, diagnostic nuclear medicine, blood chemistry tests, organ/disease panels, urinalysis, thyroid function test, other chemistry tests, other toxicology tests, blood count, immunology tests, bacterial cultures, and diagnostic mammography. ED visits were defined using procedure group codes provided in the MarketScan® data that corresponded to “emergency department visits” and “ER visits, other” or place of service codes corresponding to “emergency room – hospital”. Urgent care visits were defined using place of service categories provided in the MarketScan® data corresponding to “urgent care facility”. Inpatient stays were defined using claims from the MarketScan® inpatient admissions tables.

Table A3: Goodman-Bacon Decompositions for Clinician/Service Type Outcomes

DD Comparison	DD Estimate	Weight
PCP Visit		
Timing Groups	1.47	0.071
Never Adopters	3.36	0.853
Time-Varying Covariates	-6.74	0.077
Number of PCP Visits		
Timing Groups	0.052	0.071
Never Adopters	-0.049	0.853
Time-Varying Covariates	-0.209	0.077
Specialist Visit		
Timing Groups	-1.18	0.071
Never Adopters	1.24	0.853
Time-Varying Covariates	6.14	0.077
Number of Specialist Visits		
Timing Groups	-0.123	0.071
Never Adopters	-0.024	0.853
Time-Varying Covariates	-0.005	0.077
Preventive Visit		
Timing Groups	-0.486	0.071
Never Adopters	-0.680	0.853
Time-Varying Covariates	-1.57	0.077
Number of Preventive Visits		
Timing Groups	0.005	0.071
Never Adopters	-0.000	0.853
Time-Varying Covariates	-0.027	0.077
Diagnostic Visit		
Timing Groups	1.16	0.071
Never Adopters	1.76	0.853
Time-Varying Covariates	-1.31	0.077
Number of Diagnositc Visits		
Timing Groups	0.071	0.071
Never Adopters	0.022	0.853
Time-Varying Covariates	-0.027	0.077

Notes: "Timing Groups" indicates that the control group in the TWFE comparison includes both MSAs that never adopt PSL mandates during our sample period and those that do adopt prior to their adoption. "Never Adopters" indicates that the control group in the TWFE comparison is restricted to MSAs that never adopt PSL mandates during our sample period. "Time-Varying Covariates" indicates the influence of time-varying covariates on the TWFE estimates.

Table A4: Goodman-Bacon Decompositions for Clinical Setting Outcomes

DD Comparison	DD Estimate	Weight
Office Visit		
Timing Groups	1.87	0.071
Never Adopters	3.37	0.853
Time-Varying Covariates	-5.77	0.077
Number of Office Visits		
Timing Groups	-0.011	0.071
Never Adopters	-0.131	0.853
Time-Varying Covariates	0.134	0.077
Emergency Department Visit		
Timing Groups	0.042	0.071
Never Adopters	-0.018	0.853
Time-Varying Covariates	-1.91	0.077
Number of Emergency Department Visits		
Timing Groups	-0.018	0.071
Never Adopters	-0.021	0.853
Time-Varying Covariates	-0.053	0.077
Urgent Care Visit		
Timing Groups	-0.979	0.071
Never Adopters	-0.515	0.853
Time-Varying Covariates	2.18	0.077
Number of Urgent Care Visits		
Timing Groups	0.020	0.071
Never Adopters	-0.060	0.853
Time-Varying Covariates	0.100	0.077
Hospital Stay		
Timing Groups	0.081	0.071
Never Adopters	0.003	0.853
Time-Varying Covariates	0.015	0.077
Number of Hospital Stays		
Timing Groups	-0.026	0.071
Never Adopters	-0.005	0.853
Time-Varying Covariates	0.014	0.077

Notes: "Timing Groups" indicates that the control group in the TWFE comparison includes both MSAs that never adopt PSL mandates during our sample period and those that do adopt prior to their adoption. "Never Adopters" indicates that the control group in the TWFE comparison is restricted to MSAs that never adopt PSL mandates during our sample period. "Time-Varying Covariates" indicates the influence of time-varying covariates on the TWFE estimates.

Figure A5: Two-Way Fixed Effect Event Study Estimates of the Impact of PSL Mandates on Health Care Utilization by Clinician/Service Type

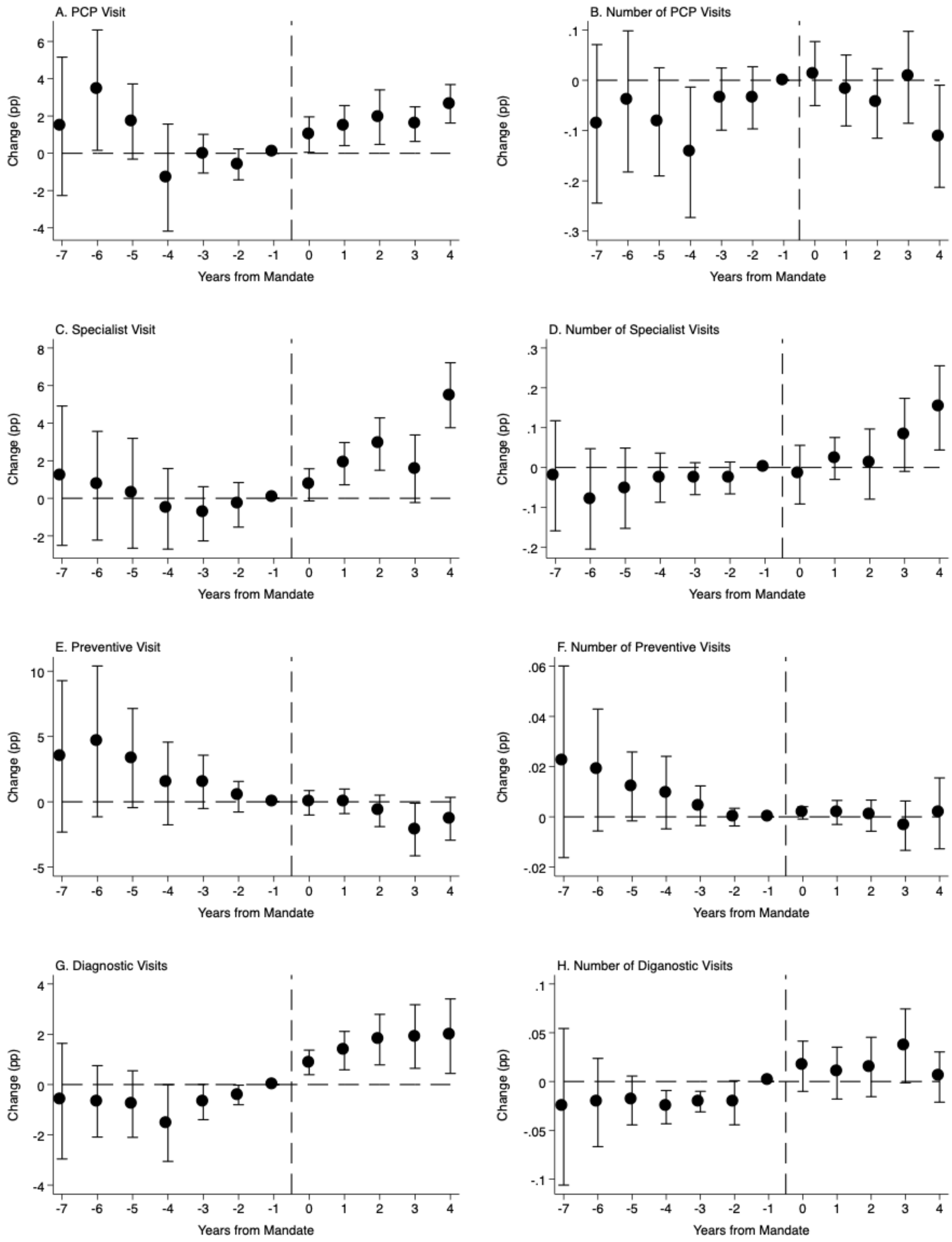

Notes: Event study estimates were calculated from difference-in-differences models estimated using two-way fixed effects and include controls for MSA-by-year age, sex, race and ethnicity composition, educational attainment, state-level share uninsured and unemployment rates, and year and MSA-by-state fixed effects.. The vertical dashed line indicates the timing of PSL mandate exposure. The unit of observation is the MSA-by-state-by-year and standard errors clustered at the state level.

Figure A6: Two-Way Fixed Effect Event Study Estimates of the Impact of PSL Mandates on Health Care Utilization by Clinical Setting

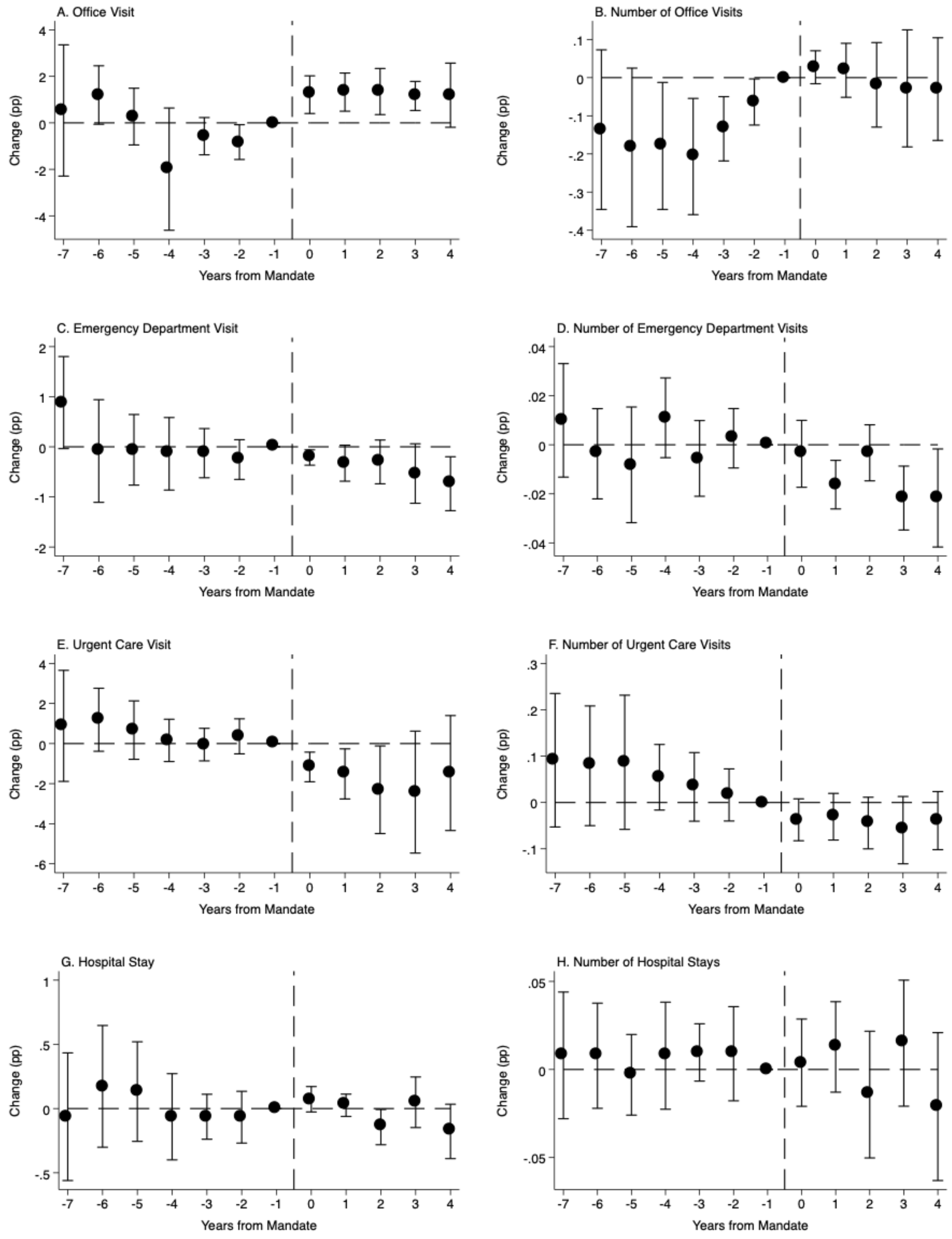

Notes: Event study estimates were calculated from difference-in-differences models estimated using two-way fixed effects and include controls for MSA-by-year age, sex, race and ethnicity composition, educational attainment, state-level share uninsured and unemployment rates, and year and MSA-by-state fixed effects. The vertical dashed line indicates the timing of PSL mandate exposure. The unit of observation is the MSA-by-state-by-year and standard errors clustered at the state level.

Table A7: Two-Way Fixed Effect Estimates of the Impact of PSL Mandates on Health Care Utilization by Clinician/Service Type, 2011 – 2019

	Exposed MSAs		Unexposed MSAs			
Outcome	Pre-Mandate	Post-Mandate	Pre-Mandate	Post-Mandate	Unadjusted DD Estimate	TWFE DD Estimate (standard error)
	(1)	(2)	(3)	(4)	(5)	(6)
PCP Visit (%)	72.12	74.40	75.09	75.32	2.05	1.85** (0.781)
Number of PCP Visits	3.05	2.97	3.19	3.01	0.104	0.024 (0.034)
Specialist Visit (%)	41.57	46.22	44.59	48.20	1.04	2.24*** (0.772)
Number of Specialist Visits	3.08	3.23	3.19	3.27	0.065	0.037 (0.040)
Preventive Visit (%)	36.93	42.59	38.34	46.28	-2.29	-1.06 (1.04)
Number of Preventive Visits	1.09	1.11	1.11	1.13	-0.012	-0.001 (0.003)
Diagnostic Visit (%)	30.79	33.48	35.04	35.66	2.06	1.86*** (0.561)
Number of Diagnostic Visits	1.87	2.02	1.91	2.02	0.033	0.029*** (0.010)

Notes: Unadjusted DD estimates were calculated as the outcome difference between the post- and pre-mandate periods for the unexposed MSAs subtracted from the difference between the post- and pre-mandate periods for the exposed MSAs. TWFE DD estimates were derived from difference-in-differences models estimated using two-way fixed effects and include controls for MSA-by-year age, sex, race and ethnicity composition, educational attainment, state-level share uninsured and unemployment rates, and year and MSA-by-state fixed effects. The unit of observation is the MSA-by-year, standard errors clustered at the state level, and all models are weighted by the number of sample individuals residing in each MSA multiplied by the inverse probability of treatment weights. The sample consisted of 247 MSAs, 62 of which were exposed to a PSL mandate, observed over a 9-year period for a total of 2,223 observations.
p<0.10, p<0.05**, p<0.01****

Table A8: Two-Way Fixed Effect Estimates of the Impact of PSL Mandates on Health Care Utilization by Clinical Setting, 2011 – 2019

	Exposed MSAs		Unexposed MSAs			
Outcome	Pre-Mandate	Post-Mandate	Pre-Mandate	Post-Mandate	Unadjusted DD Estimate	TWFE DD Estimate (standard error)
	(1)	(2)	(3)	(4)	(5)	(6)
Office Visit (%)	79.05	82.16	82.60	84.35	1.36	1.95**<br>(0.741)
Number of Office Visits	4.57	4.76	4.93	5.01	0.110	0.090*<br>(0.045)
Emergency Department Visit (%)	9.75	9.83	11.51	11.70	-0.110	-0.192<br>(0.230)
Number of Emergency Department Visits	1.26	1.26	1.31	1.32	-0.007	-0.012*<br>(0.006)
Urgent Care Visit (%)	6.15	8.22	6.90	11.62	-2.66	-1.73*<br>(0.861)
Number of Urgent Care Visits	1.40	1.41	1.40	1.48	-0.067	-0.059**<br>(0.028)
Hospital Stay (%)	3.58	3.38	4.07	3.77	0.100	0.037<br>(0.066)
Number of Hospital Stays	1.25	1.28	1.26	1.29	-0.000	-0.004<br>(0.007)

Notes: Unadjusted DD estimates were calculated as the outcome difference between the post- and pre-mandate periods for the unexposed MSAs subtracted from the difference between the post- and pre-mandate periods for the exposed MSAs. TWFE DD estimates were derived from difference-in-differences models estimated using two-way fixed effects and include controls for MSA-by-year age, sex, race and ethnicity composition, educational attainment, state-level share uninsured and unemployment rates, and year and MSA-by-state fixed effects. The unit of observation is the MSA-by-year, standard errors clustered at the state level, and all models are weighted by the number of sample individuals residing in each MSA multiplied by the inverse probability of treatment weights. The sample consisted of 247 MSAs, 62 of which were exposed to a PSL mandate, observed over a 9-year period for a total of 2,223 observations.
p<0.10, p<0.05**, p<0.01****

Table A9: Estimates of the Impact of PSL Mandates on Health Care Utilization by Clinician/Service Type – Low PSL Sample, 2011 – 2019

	Exposed MSAs		Unexposed MSAs		Regression Estimates		
Outcome	Pre-Mandate	Post-Mandate	Pre-Mandate	Post-Mandate	Unadjusted DD Estimate	C&S Estimate (standard error)	TWFE Estimate (standard error)
	(1)	(2)	(3)	(4)	(5)	(6)	(7)
PCP Visit (%)	69.19	74.93	76.84	74.09	8.49	6.46**<br>(3.03)	4.98***<br>(1.15)
Number of PCP Visits	3.19	3.21	3.38	2.93	0.466	0.027<br>(0.063)	0.280*<br>(0.158)
Specialist Visit (%)	35.45	38.52	44.40	48.74	-1.27	2.81<br>(2.01)	1.09<br>(1.50)
Number of Specialist Visits	3.00	3.09	3.30	3.05	0.346	0.124<br>(0.119)	0.028<br>(0.055)
Preventive Visit (%)	34.18	39.54	35.53	36.40	4.49	4.72*<br>(2.52)	3.14<br>(1.98)
Number of Preventive Visits	1.09	1.10	1.11	1.11	0.012	0.034<br>(0.026)	-0.011**<br>(0.005)
Diagnostic Visit (%)	27.56	30.79	35.42	36.88	1.77	2.17***<br>(0.775)	3.54***<br>(1.24)
Number of Diagnostic Visits	1.85	2.01	1.95	1.92	0.181	-0.014<br>(0.042)	0.081***<br>(0.029)

Notes: Unadjusted DD estimates were calculated as the outcome difference between the post- and pre-mandate periods for the unexposed MSAs subtracted from the difference between the post- and pre-mandate periods for the exposed MSAs. Adjusted DD estimates were derived from difference-in-differences models estimated using two-way fixed effects and include controls for MSA-by-year age, sex, race and ethnicity composition, educational attainment, state-level share uninsured and unemployment rates, and year and MSA-by-state fixed effects. C&S estimates were derived from the Callaway and Sant'Anna procedure for staggered intervention timing and use stabilized inverse probability weighting for improved covariate balance. The unit of observation is the MSA-by-year, standard errors clustered at the state level, and all models are weighted by the number of sample individuals residing in each MSA multiplied by the inverse probability of treatment weights. The sample consisted of 247 MSAs, 62 of which were exposed to a PSL mandate, observed over a 9-year period for a total of 2,223 observations.
p<0.10, p<0.05**, p<0.01****

Table A10: Estimates of the Impact of PSL Mandates on Health Care Utilization by Clinical Setting – Low PSL Sample, 2011 – 2019

	Exposed MSAs		Unexposed MSAs		Regression Estimates		
Outcome	Pre-Mandate	Post-Mandate	Pre-Mandate	Post-Mandate	Unadjusted DD Estimate	C&S Estimate (standard error)	TWFE Estimate (standard error)
	(1)	(2)	(3)	(4)	(5)	(6)	(7)
Office Visit (%)	74.84	80.61	83.42	84.75	4.43	7.18*** (2.60)	4.07*** (1.13)
Number of Office Visits	4.50	4.64	5.18	4.93	0.384	0.055 (0.091)	0.195*** (0.068)
Emergency Department Visit (%)	9.50	10.51	10.95	11.48	0.467	1.33** (0.616)	-0.079 (0.809)
Number of Emergency Department Visits	1.26	1.27	1.32	1.23	0.092	0.083 (0.056)	0.017 (0.026)
Urgent Care Visit (%)	6.84	9.03	6.75	11.39	-2.46	0.433 (1.65)	-3.02*** (0.974)
Number of Urgent Care Visits	1.43	1.47	1.41	1.48	-0.021	-0.008 (0.065)	0.000 (0.047)
Hospital Stay (%)	3.40	3.19	3.69	3.62	-0.130	-0.438 (0.319)	0.303 (0.214)
Number of Hospital Stays	1.23	1.26	1.21	1.21	0.033	0.046 (0.030)	0.007 (0.013)

Notes: Unadjusted DD estimates were calculated as the outcome difference between the post- and pre-mandate periods for the unexposed MSAs subtracted from the difference between the post- and pre-mandate periods for the exposed MSAs. Adjusted DD estimates were derived from difference-in-differences models estimated using two-way fixed effects and include controls for MSA-by-year age, sex, race and ethnicity composition, educational attainment, state-level share uninsured and unemployment rates, and year and MSA-by-state fixed effects. C&S estimates were derived from the Callaway and Sant'Anna procedure for staggered intervention timing and use stabilized inverse probability weighting for improved covariate balance. The unit of observation is the MSA-by-year, standard errors clustered at the state level, and all models are weighted by the number of sample individuals residing in each MSA multiplied by the inverse probability of treatment weights. The sample consisted of 247 MSAs, 62 of which were exposed to a PSL mandate, observed over a 9-year period for a total of 2,223 observations.
p<0.10, p<0.05**, p<0.01****

Table A11: Individual-Level Callaway and Sant'Anna Estimates of the Impact of PSL Mandates on Health Care Utilization by Clinician/Service Type, 2011 – 2019

	Full Sample		Low PSL Sample	
	(1)	(2)	(3)	(4)
**PCP Visit**	4.79***	2.64**	6.46**	7.03***
	(1.74)	(1.16)	(3.03)	(1.63)
Baseline Mean (%)	72.12	74.64	69.19	73.08
Observations	2,223	18,852,582	2,223	6,418,477
**Number of PCP Visits**	-0.001	0.201**	0.027	0.343*
	(0.046)	(0.090)	(0.063)	(0.176)
Baseline Mean	3.05	3.03	3.19	3.14
Observations	2,223	14,156,746	2,223	4,753,251
**Specialist Visit**	2.71***	3.08	2.81	6.64
	(0.882)	(2.06)	(2.01)	(4.11)
Baseline Mean (%)	41.57	44.32	35.45	40.27
Observations	2,223	18,852,582	2,223	6,418,477
**Number of Specialist Visits**	0.171***	0.170	0.124	0.262*
	(0.058)	(0.122)	(0.119)	(0.134)
Baseline Mean	3.08	3.08	3.00	3.03
Observations	2,223	8,751,966	2,223	2,698,806
**Preventive Visit**	2.75*	6.87***	4.72*	9.28***
	(1.59)	(2.01)	(2.52)	(3.09)
Baseline Mean (%)	36.93	38.27	34.18	38.04
Observations	2,223	18,852,582	2,223	6,418,477
**Number of Preventive Visits**	0.028*	-0.006	0.034	-0.008
	(0.015)	(0.011)	(0.026)	(0.013)
Baseline Mean	1.09	1.11	1.09	1.12
Observations	2,223	7,775,444	2,223	2,627,798
**Diagnostic Visit**	2.20***	2.62*	2.17***	5.41***
	(0.505)	(1.34)	(0.775)	(1.31)
Baseline Mean (%)	30.79	32.90	27.56	30.45
Observations	2,223	18,852,582	2,223	6,418,477
**Number of Diagnostic Visits**	0.078**	0.031	-0.014	-0.050**
	(0.036)	(0.024)	(0.042)	(0.024)
Baseline Mean	1.87	1.863	1.85	1.84
Observations	2,223	6,389,482	2,223	2,016,791

Notes: Columns 1 and 3 replicate MSA-level estimates from Table 2 and Table A7. All estimates were derived from the Callaway and Sant'Anna procedure for staggered intervention timing and use stabilized inverse probability weighting for improved covariate balance. Covariates included MSA age, sex, race and ethnicity composition, educational attainment, and state-level share uninsured and unemployment rates. The unit of observation in Columns 2 and 4 is the individual and standard errors clustered at the state level. *$p<0.10$*, $p<0.05$**, $p<0.01$****

Table A12: Individual-Level Callaway and Sant'Anna Estimates of the Impact of PSL Mandates on Health Care Utilization by Clinical Setting, 2011 – 2019

	Full Sample		Low PSL Sample	
	(1)	(2)	(3)	(4)
**Office Visit**	4.63***	3.20***	7.18***	5.60**
	(1.66)	(1.12)	(2.60)	(2.16)
Baseline Mean (%)	79.05	81.83	74.84	79.60
Observations	2,223	18,852,582	2,223	6,418,477
**Number of Office Visits**	0.031	0.257*	0.055	0.344*
	(0.069)	(0.154)	(0.091)	(0.185)
Baseline Mean	4.57	4.64	4.50	4.62
Observations	2,223	15,642,793	2,223	5,195,330
**Emergency Department Visit**	0.516	-0.923*	1.33**	0.412
	(0.316)	(0.559)	(0.616)	(0.706)
Baseline Mean (%)	9.75	10.99	9.50	11.02
Observations	2,223	18,852,582	2,223	6,418,477
**Number of Emergency Department Visits**	0.065	0.049***	0.083	-0.025
	(0.040)	(0.013)	(0.056)	(0.019)
Baseline Mean	1.26	1.30	1.26	1.30
Observations	2,223	2,140,605	2,223	741,609
**Urgent Care Visits**	-1.79	-2.08*	0.433	-2.12
	(1.22)	(1.25)	(1.65)	(1.47)
Baseline Mean (%)	1.40	6.38	6.84	7.17
Observations	2,223	18,852,582	2,223	6,418,477
**Number of Urgent Care Visits**	0.102**	0.033	-0.008	0.053
	(0.042)	(0.069)	(0.065)	(0.185)
Baseline Mean	1.40	1.41	1.43	1.45
Observations	2,223	1,424,046	2,223	540,976
**Hospital Stay**	0.346**	0.239	-0.438	-0.446
	(0.162)	(0.302)	(0.319)	(0.340)
Baseline Mean (%)	3.58	3.90	3.40	3.75
Observations	2,223	18,852,582	2,223	6,418,477
**Number of Hospital Stays**	0.033	0.013	0.046	0.016
	(0.030)	(0.014)	(0.030)	(0.020)
Baseline Mean	1.25	1.252	1.23	1.23
Observations	2,223	731,077	2,223	239,330

Notes: Columns 1 and 3 replicate MSA-level estimates from Table 3 and Table A8. All estimates were derived from the Callaway and Sant'Anna procedure for staggered intervention timing and use stabilized inverse probability weighting for improved covariate balance. Covariates included MSA age, sex, race and ethnicity composition, educational attainment, and state-level share uninsured and unemployment rates. The unit of observation in Columns 2 and 4 is the individual and standard errors clustered at the state level. *p<0.10*, p<0.05**, p<0.01****

Table A13: Individual-Level Two-Way Fixed Effects Estimates of the Impact of PSL Mandates on Health Care Utilization by Clinician/Service Type, 2011 – 2019

	Full Sample			Low PSL Sample		
	(1)	(2)	(3)	(4)	(5)	(6)
**PCP Visit**	1.85**	1.59	1.47	4.98***	4.61***	4.32***
	(0.781)	(1.07)	(1.16)	(1.15)	(1.70)	(1.83)
Individual Fixed Effects	No	No	Yes	No	No	Yes
Baseline Mean (%)	72.12	74.64	74.64	69.19	73.08	73.08
Observations	2,223	18,852,582	18,852,582	2,223	6,418,477	6,418,477
**Number of PCP Visits**	0.024	0.056	0.031	0.280*	0.012	0.044
	(0.034)	(0.035)	(0.060)	(0.158)	(0.063)	(0.084)
Individual Fixed Effects	No	No	Yes	No	No	Yes
Baseline Mean	3.05	3.03	3.03	3.19	3.14	3.14
Observations	2,223	14,156,746	14,156,746	2,223	4,753,251	4,753,251
**Specialist Visit**	2.24***	2.05**	2.84***	1.09	2.61	2.77
	(0.772)	(0.775)	(0.867)	(1.50)	(1.72)	(1.84)
Individual Fixed Effects	No	No	Yes	No	No	Yes
Baseline Mean (%)	41.57	44.32	44.32	35.45	40.266	40.266
Observations	2,223	18,852,582	18,852,582	2,223	6,418,477	6,418,477
**Number of Specialist Visits**	0.037	0.015	0.014	0.028	0.051	0.018
	(0.040)	(0.027)	(0.041)	(0.055)	(0.040)	(0.059)
Individual Fixed Effects	No	No	Yes	No	No	Yes
Baseline Mean	3.08	3.08	3.08	3.00	3.03	3.03
Observations	2,223	8,751,966	8,751,966	2,223	2,698,806	2,698,806
**Preventive Visit**	-1.06	0.333	0.419	3.14	5.63***	4.67***
	(1.04)	(0.590)	(0.469)	(1.98)	(0.608)	(0.728)
Individual Fixed Effects	No	No	Yes	No	No	Yes
Baseline Mean (%)	36.93	38.27	38.27	34.18	38.04	38.04
Observations	2,223	18,852,582	18,852,582	2,223	6,418,477	6,418,477
**Number of Preventive Visits**	-0.001	-0.002	-0.006	-0.011	-0.012**	-0.011
	(0.003)	(0.005)	(0.006)	(0.005)	(0.006)	(0.008)
Individual Fixed Effects	No	No	Yes	No	No	Yes
Baseline Mean	1.09	1.11	1.11	1.09	1.12	1.12
Observations	2,223	7,775,444	7,775,444	2,223	2,627,798	2,627,798
**Diagnostic Visit**	1.86***	1.275***	1.730***	3.54***	2.11***	2.67***
	(0.561)	(0.462)	(0.457)	(1.24)	(0.580)	(0.728)
Individual Fixed Effects	No	No	Yes	No	No	Yes
Baseline Mean (%)	30.79	32.90	32.90	27.56	30.45	30.45
Observations	2,223	18,852,582	18,852,582	2,223	6,418,477	6,418,477
**Number of Diagnostic Visits**	0.029***	0.011	0.017	0.081***	0.011	-0.011
	(0.010)	(0.013)	(0.018)	(0.029)	(0.011)	(0.016)
Individual Fixed Effects	No	No	Yes	No	No	Yes
Baseline Mean	1.87	1.86	1.86	1.85	1.84	1.84
Observations	2,223	6,389,482	6,389,482	2,223	2,016,791	2,016,791

Notes: Columns 1 and 4 replicate MSA-level estimates from Table 2 and Table A7. All estimates were derived from difference-in-differences models estimated using two-way fixed effects and included controls for MSA-by-year age, sex, race and ethnicity composition, educational attainment, state-level share uninsured and unemployment rates, and year and MSA-by-state fixed effects. The unit of observation in Columns 2, 3, 5, and 6 is the individual and standard errors clustered at the state level. *p<0.10*, p<0.05**, p<0.01****

Table A14: Individual-Level Two-Way Fixed Effects Estimates of the Impact of PSL Mandates on Health Care Utilization by Clinical Setting, 2011 – 2019

	**Full Sample**			**Low PSL Sample**		
	(1)	(2)	(3)	(4)	(5)	(6)
**Office Visit**	1.95**	1.74*	1.70	4.07***	4.93***	3.74**
	(0.741)	(0.951)	(1.054)	(1.13)	(1.70)	(1.85)
Individual Fixed Effects	No	No	Yes	No	No	Yes
Baseline Mean (%)	79.05	81.83	81.83	74.84	79.60	79.60
Observations	2,223	18,852,582	18,852,582	2,223	6,418,477	6,418,477
**Number of Office Visits**	0.090*	0.050*	0.043	0.195***	0.140	0.173
	(0.045)	(0.028)	(0.056)	(0.068)	(0.050)	(0.065)
Individual Fixed Effects	No	No	Yes	No	No	Yes
Baseline Mean	4.57	4.64	4.64	4.50	4.62	4.62
Observations	2,223	15,642,793	15,642,793	2,223	5,195,330	5,195,330
**Emergency Department Visits**	-0.192	-0.398*	-0.406	-0.079	-0.027	-0.112
	(0.230)	(0.217)	(0.273)	(0.809)	(0.499)	(0.622)
Individual Fixed Effects	No	No	Yes	No	No	Yes
Baseline Mean (%)	9.75	10.99	10.99	9.50	11.02	11.02
Observations	2,223	18,852,582	18,852,582	2,223	6,418,477	6,418,477
**Number of Emergency Department Visits**	-0.012*	-0.018**	-0.026	0.017	-0.030***	-0.035
	(0.006)	(0.008)	(0.017)	(0.026)	(0.010)	(0.023)
Individual Fixed Effects	No	No	Yes	No	No	Yes
Baseline Mean	1.26	1.30	1.30	1.26	1.30	1.30
Observations	2,223	2,140,605	2,140,605	2,223	741,609	741,609
**Urgent Care Visits**	-1.73*	-0.474	-0.012	-3.02***	-1.30*	-1.49*
	(0.861)	(0.558)	(0.494)	(0.974)	(0.716)	(0.802)
Individual Fixed Effects	No	No	Yes	No	No	Yes
Baseline Mean (%)	6.15	6.38	6.38	6.84	7.17	7.17
Observations	2,223	18,852,582	18,852,582	2,223	6,418,477	6,418,477
**Number of Urgent Care Visits**	-0.059**	-0.036**	-0.038	0.000	-0.039	-0.116
	(0.028)	(0.014)	(0.035)	(0.047)	(0.027)	(0.090)
Individual Fixed Effects	No	No	Yes	No	No	Yes
Baseline Mean	1.40	1.41	1.41	1.43	1.450	1.450
Observations	2,223	1,424,046	1,424,046	2,223	540,976	540,976
**Hospital Stay**	0.037	0.040	0.027	0.303	0.052	0.020
	(0.066)	(0.077)	(0.068)	(0.214)	(0.056)	(0.074)
Individual Fixed Effects	No	No	Yes	No	No	Yes
Baseline Mean (%)	3.58	3.90	3.90	3.40	3.75	3.75
Observations	2,223	18,852,582	18,852,582	2,223	6,418,477	6,418,477
**Number of Hospital Stays**	-0.004	-0.005	-0.009	0.007	0.001	-0.004
	(0.007)	(0.006)	(0.010)	(0.013)	(0.008)	(0.012)
Individual Fixed Effects	No	No	Yes	No	No	Yes
Baseline Mean	1.25	1.252	1.252	1.23	1.23	1.23
Observations	2,223	731,077	731,077	2,223	239,330	239,330

Notes: Columns 1 and 4 replicate MSA-level estimates from Table 3 and Table A8. All estimates were derived from difference-in-differences models estimated using two-way fixed effects and included controls for MSA-by-year age, sex, race and ethnicity composition, educational attainment, state-level share uninsured and unemployment rates, and year and MSA-by-state fixed effects. The unit of observation in Columns 2, 3, 5, and 6 is the individual and standard errors clustered at the state level. *p<0.10*, p<0.05**, p<0.01****

Table A15: Demographic Characteristic Changes Associated with PSL Mandates

	**Regression Estimates**	**Percentage Change**
	(1)	(2)
Female	0.765<br>(1.144)	1.63
Age	0.110*<br>(0.056)	0.21
Non-Hispanic Black	-0.281**<br>(0.134)	5.39
Hispanic	0.164<br>(0.267)	1.15
Other Race or Ethnicity	0.117<br>(0.158)	1.65
Non-Hispanic White	-0.001<br>(0.314)	0.00
Less than High School	0.148**<br>(0.055)	2.33
High School	0.004*<br>(0.002)	0.01
Some College	-0.801***<br>(0.185)	3.01
College	0.294<br>(0.200)	0.83
State Unemployment Rate	0.620*<br>(0.316)	7.98
Uninsurance Rate	-0.825<br>(0.963)	5.49
MSA-Years	2,223	

Notes: Estimates were derived from difference-in-differences models estimated using two-way fixed effects models and include controls for MSA-by-year age, sex, race and ethnicity composition, educational attainment, state-level share uninsured and unemployment rates, and year and MSA-by-state fixed effects. Covariates were excluded from the model when they were used as the dependent variable (e.g., regressions with educational attainment as the dependent variables excluded educational attainment covariates). Each row contains the estimate of the association between PSL mandates and the listed outcome from a separate regression and standard errors in each regression are clustered at the state level. *p<0.10*, p<0.05**, p<0.01****

Table A16: STROBE Statement

	Item No.	Recommendation	Page No.
**Title and Abstract**	1	a. Indicate the study's design with a commonly used term in the title or abstract<br>b. Provide in the abstract an informative and balanced summary of what was done and what was found	
**Introduction**			
Background/rationale	2	Explain the scientific background and rationale for the investigation being reported	3
Objectives	3	State specific objectives, including any prespecified by hypotheses	4
**Methods**			
Study design	4	Present key elements of study design early in the paper	4
Setting	5	Describe the setting, locations, and relevant dates, including periods of recruitment, exposure, follow-up, and data collection	4-5
Participants	6	a. Give the eligibility criteria, and the sources and methods of selection of participants. Describe methods of follow-up<br>b. For matched studies, give matching criteria and number of exposed and unexposed	a. 4<br>b. 6-7
Variables	7	Clearly define all outcomes, exposures, predictors, potential confounders, and effect modifiers. Give diagnostic criteria, if applicable	5-6
Data sources/measurement	8	For each variable of interest, give sources of data and details of methods of assessment (measurement). Describe comparability of assessment methods if there is more than one group	4-5
Bias	9	Describe any efforts to address potential sources of bias	6-8
Study size	10	Explain how the study size was arrived at	4-5
Quantitative variables	11	Explain how quantitative variables were handled in the analyses. If applicable, describe which groupings were chosen and why	4-5
Statistical methods	12	a. Describe all statistical methods, including those used to control for confounding	a. 6-8<br>b. 8

		b. Describe any methods used to examine subgroups and interactions	
**Results**			
Participants	13	a. Report numbers of individuals at each stage of study-eg numbers potentially eligible, examined for eligibility, confirmed eligible, included in the study, completing follow-up, and analyzed<br>b. Give reasons for non-participation at each stage<br>c. Consider use of a flow diagram	a. 4-5<br>b. N/A<br>c. N/A
Descriptive data	14	a. Give characteristics of study participants (eg demographic, clinical, social) and information on exposures and potential confounders<br>b. Indicate number of participants with missing data for each variable of interest<br>c. Summarize follow-up time (eg, average and total amount)	a. 9, Table 1<br>b. N/A<br>c. 4-5
Outcome data	15	Report numbers of outcome events or summary measures over time	Tables 2-3
Main results	16	a. Give unadjusted estimates and, if applicable, confounder-adjusted estimates and their precision (eg, 95% confidence interval). Make clear which confounders were adjusted for and why they were included<br>b. Report category boundaries when continuous variables were categorized<br>c. If relevant, consider translating estimates of relative risk into absolute risk for a meaningful time period	a. Tables 2-3<br>b. N/A<br>c. 10-11
Other analyses	17	Report other analyses done-eg analyses of subgroups and interactions, and sensitivity analyses	11-12
**Discussion**			
Key results	18	Summarize key results with reference to study objectives	12
Limitations	19	Discuss limitations of the study, taking into account sources of potential bias or imprecision. Discuss both direction and magnitude of any potential bias	14
Interpretation	20	Give a cautious overall interpretation of results considering objectives, limitations, multiplicity of analyses, results from similar studies, and other relevant evidence	13, 15

Generalizability	21	Discuss the generalizability (external validity) of the study results	13-14
Other Information			
Funding	22	Give the source of funding and the role of the funders for the present study and, if applicable, for the original study on which the present article is based	16

References

1. Goodman-Bacon A. Difference-in-differences with variation in treatment timing. *Journal of Econometrics.* 2021;225(2):254-277.
2. Sun L, Abraham S. Estimating dynamic treatment effects in event studies with heterogeneous treatment effects. *Journal of Econometrics.* 2021;225(2):175-199.
3. De Chaisemartin C, d'Haultfoeuille X. Two-way fixed effects estimators with heterogeneous treatment effects. *American Economic Review.* 2020;110(9):2964-2996.
4. Callaway B, Sant'Anna PH. Difference-in-differences with multiple time periods. *Journal of Econometrics.* 2021;225(2):200-230.
5. Daw JR, Hatfield LA. Matching and regression to the mean in difference-in-differences analysis. *Health services research.* 2018;53(6):4138-4156.
6. Leite WL, Jimenez F, Kaya Y, Stapleton LM, MacInnes JW, Sandbach R. An evaluation of weighting methods based on propensity scores to reduce selection bias in multilevel observational studies. *Multivariate behavioral research.* 2015;50(3):265-284.
7. Bishop CD, Leite WL, Snyder PA. Using propensity score weighting to reduce selection bias in large-scale data sets. *Journal of Early Intervention.* 2018;40(4):347-362.
